# Routine management of locally advanced cervical cancer with concurrent radiation and cisplatin. Five-year results

**DOI:** 10.1186/1472-6874-6-3

**Published:** 2006-02-07

**Authors:** Lucely Cetina, Lesbia Rivera, José Hinojosa, Adela Poitevin, Jesús Uribe, Carlos López-Graniel, David Cantú, Myrna Candelaria, Jaime de la Garza, Alfonso Dueñas-González

**Affiliations:** 1 Division of Clinical Research, Instituto Nacional de Cancerología (INCan), Mexico City, Mexico; 2 Division of Radiation Oncology, INCan, Mexico City, Mexico; 3 Division of Surgical Oncology, INCan, Mexico City, Mexico; 4 Unit of Biomedical Research on Cancer, Instituto de Investigaciones Biomédicas (IIB), Universidad Nacional Autónoma de México (UNAM)/INCan, Mexico City, México

## Abstract

**Background:**

Globally, cervical cancer primarily affects socially disadvantaged women. Five randomized trials were the foundation for adopting cisplatin-based chemotherapy during radiation as the standard of care for high-risk patients after primary radical hysterectomy who require adjuvant radiation and for locally advanced patients treated with definitive radiation. These results were obtained in clinical trials performed in carefully prepared academic centers; hence, we sought to determine whether these results could be reproduced when patients were treated on an out-of-protocol basis.

**Methods:**

We reviewed the files of 294 patients with locally advanced cervical cancer who received radiation plus weekly cisplatin as routine management between 1999 to 2003, and analyzed treatment compliance, response rate, toxicity, and survival.

**Results:**

A total of 294 patients who received radiation and cisplatin were analyzed. Mean age was 43.8 years (range, 26–68 years). The majority of cases were squamous cell carcinoma (87.8%), and distribution according to International Federation of Gynecology and Obstetrics (FIGO) stage was as follows: IB2-IIA, 23%; IIB, 53.3%, and IIIB, 23%; there were only two IVA cases. Overall, 96% of patients completed external beam, and intracavitary therapy. The majority of patients (67%) received the planned six courses of weekly cisplatin. Complete responses were achieved in 243 (83%) patients, whereas 51 (17%) had either persistent (32 patients, 10.8%) or progressive (19 patients, 6.4%) disease. At median follow-up (28 months; range, 2–68 months), 36 patients (12.2%) have relapsed (locally 30.5, and systemically, 69.5%). The most common toxicities were hematologic and gastrointestinal, in the majority of cases considered mild-moderate. At median follow-up (28 months; range, 2–68 months), overall and progression-free survival are 76.5 and 67%, respectively.

**Conclusion:**

Our results support use of chemoradiation with six weekly applications of cisplatin at 40 mg/m^2 ^during external radiation for routine management of locally advanced cervical cancer.

## Background

Cervical cancer remains one of the biggest killers of women worldwide. The epidemiology of cervical cancer is strongly related with a population's standard of living; thus, underdeveloped countries present elevated mortality rates that can be as high as >70 per 100,000 inhabitants [1]. In Mexico – as in many other countries with limited health resources – cervical cancer mortality stands at 14 per 100,000 inhabitants, and the majority of cases are diagnosed in locally advanced-disease stages IB2-IVA according to the International Federation of Gynecology and Obstetrics (FIGO) classification [2].

Although chemotherapy has been used in its neoadjuvant modality and concomitant with radiation therapy in treatment of locally advanced cervical cancer for approximately 20 years, it was not until 1999 that five randomized studies, which included nearly 2,000, patients were published, demonstrating that survival rate with radiation therapy alone was lower than with radiation therapy with concomitant chemotherapy (RT/CT) utilizing cisplatin [3-7]. Later, a meta-analysis corroborated these findings, confirming that chemoradiation offers an absolute survival benefit at 5 years of 12% [8]. Thus, cisplatin-based chemoradiation was widely accepted as the standard of care for patients with cervical cancer whose treatment required radiation.

The realization that cervical cancer primarily affects socioeconomically disadvantaged women would suggest that results obtained from clinical trials, usually performed at carefully prepared academic centers, cannot be easily reproduced in a community setting. Aside from the combined treatment's technical complexity, socially disadvantaged women may be more susceptible to the combined treatment's toxic effects due to poor nutritional status, presence of co-morbid chronic conditions, and/or difficulties in accessing medical care during treatment [9,10].

Patients with cervical cancer attending to our Institution (Instituto Nacional de Cancerología [INCan ] in Mexico City) are in general socially disadvantaged women; hence, we wanted to analyze our results of treatment with cisplatin chemoradiation, which was adopted in 1999 as routine management.

## Methods

### Patients

We conducted a retrospective review of 294 consecutive newly diagnosed and previously untreated patients who received radiotherapy and concurrent cisplatin at the INCan between January 1999 and December 2003. All patients had a histologic diagnosis of cervical carcinoma and were staged according to the FIGO classification using standard pre-treatment workup (pelvic examination was performed without administration of anesthesia) [11]. The sole situations in which cisplatin was not used for sensitization were the following: patients >70 years of age; hepatic insufficiency, and any degree of creatinine elevation (>1.2 mg/mL), as well as for patients with diabetes mellitus and high blood pressure. As this was a retrospective review on patients treated under routinely basis, no ethical approval was required by our Institution

### Treatment

Patients received external beam radiation using megavoltage machines (Co^60 ^or lineal accelerator equipment) with a minimum photon-beam energy of 2.25 MV with an isocenter technique to the whole pelvis for a total dose of 50 Gy (5 weeks, 2 Gy fractions from Monday to Friday) followed by one or two intracavitary Cesium (low-dose rate) applications within 2 weeks of finishing external radiation. The planned total dose to point A was at least 85 Gy. Patients were treated with the conventional 4-field box technique. Irradiated volume was to include the whole uterus, paracervical, parametrial, and uterosacral regions, as well as external iliac, hypogastric, and obturator lymph nodes.

Cisplatin was administered for 6 weeks during external radiation, beginning on the first day of radiation. Cisplatin infusion was administered within 2 h either before or after radiation application. A dose of 40 mg/m^2 ^(maximum dose, 80 mg) was used and administered via a peripheral vein to patients in an out-patient setting as follows: 1,000 mL of normal saline for 1 h followed by cisplatin diluted in 500 mL of normal saline containing 62.5 mL of 20% mannitol for 1 h, followed by 500 mL of normal saline for 30 min. Intravenously (i.v.), 8 mg of dexametasone and 8 mg of ondansetron were employed as antiemetic prophylaxis. Cisplatin (but no radiation) was withheld in any case involving grade 3 toxicity until the toxicity regressed to any grade of <3; in patients with grade 3 toxicity that persisted >2 weeks, chemotherapy was no longer administered. Radiation was only stopped in cases of grade 4 hematologic or non-hematologic toxicity until toxicity resolved to at least grade 3.

### Response evaluation

Response to chemoradiation was clinically and cytologically evaluated at the third month after ending treatment. Complete response was registered when no clinical and cytologic disease evidence existed; all other cases were registered as persistent or progressive disease. Persistent disease was considered with any less-than-complete response, and progression was defined as local or systemic: local existed when there was an increase >25% in initial lesion size, and systemic was considered with the appearance of new lesions irrespective of local response.

### Evaluation of toxicity

Acute and chronic toxicities to chemoradiation were evaluated according to National Cancer Institute (NCI) common toxicity criteria. During treatment, blood counts and chemistry profiles were performed prior to each cisplatin administration.

### Follow-up

Upon treatment completion, patients were evaluated every 3 months for the first year, every 4 months during the second year, every 6 months during the third year, and annually thereafter. At each visit, a physical and pelvic examination, blood counts, clinical chemistry, and chest x-rays were performed. Computed tomography (CT) scan, ultrasound (US), and other imaging studies were conducted when appropriate. Suspected cases of persistent or recurrent disease were confirmed by biopsy whenever possible.

### Statistical analysis

Overall and progression-free survival times were analyzed on an intention-to-treat basis and were registered from date of diagnosis to date of death or date of last visit, and from date of diagnosis to date of progression or relapse respectively. Curves were constructed using the Kaplan-Meier method [12].

## Results

### Patient characteristics

A total of 294 patients who received radiation and cisplatin were analyzed. Patient clinical characteristics are shown in Table 1. Mean age was 43.8 years (range, 26–68 years), the majority of cases were squamous cell carcinoma (87.8%), and distribution according to FIGO stage was IB2-IIA 23.2%, IIB 53.4%, and IIIB, 20.4%; there were only two IVA cases. Mean hemoglobin at diagnosis was 12.7 g/dL with ranges between 4.4 and 18.2 g/dL.

**Table 1:** Clinical characteristics (294 patients)

Characteristics	Number (%)
Mean age (years)	43.8 (26–68)
Histology	
Squamous	258 (87.8)
Adenocarcinoma	21 (7.1)
Adenosquamous	10 (3.4)
Glassy cell	1 (0.3)
Papillary	4 (1.4)
Mean hemoglobin at diagnosis	12.7 (4.4–18.2)

Stage distribution is shown in Table 5.

### Treatment

With the exception of one patient who only received 16 Gy, all patients completed external beam radiation therapy and chemotherapy. Mean dose of external beam radiation was 50.45 Gy (50–64 Gy). Brachytherapy insertions to complete the planned dose were applied once in 249 patients (84.3%) and twice in 33 (11.2%). Overall, 96% of patients completed both phases, including external beam and intracavitary therapy. Twelve (4%) patients did not receive brachytherapy: one abandoned treatment during external radiation, and 11 abandoned treatment due to the fact that disease persistence conditioned technical difficulties for insertions. Mean dose to point A was 81.3 (Gy, 80.8–9.48) and overall treatment time was 53 days (range, 40–82 days) (Table 2).

**Table 2:** Radiation treatment

	Number (%)
External beam radiation*	293 (99.6%)
External beam radiotherapy + brachytherapy**	282 (96%)
Mean dose external beam (Gy)	50.45 (50–64)
Mean dose point A (Gy)	81.3 (80.8–90.4)
Overall treatment time (days)	53 (40–82)

*One patient received only 16 Gy of external radiation prior to abandoning treatment; **11 cases did not complete brachytherapy due to technical reasons related with persistent voluminous disease.

With regard to chemotherapy, Table 3 demonstrates that the majority of patients (197, 67.0%) received the six planned cycles, 64 patients (21.7%) were administered five cycles, and 24 (8.1%) had only four cycles. There were seven (2.3%) and two patients (0.7%) who could only receive three and two applications, respectively, while two patients received seven applications of cisplatin.

**Table 3:** Chemotherapy delivered

Weekly cycles	Number of patients (%)
6	197 (67)
5	64 (21.7)
4	24 (8.1)
3	(2.3)
2	(0.8)

### Treatment response

Treatment response was evaluated by intention-to-treat. Complete responses were achieved in 243 (83%) patients, whereas 51 (17%) patients had either persistent (32 patients, 10.8%) or progressive (19 patients 6.4%) disease. Among patients with progressive disease, all had systemic progression, and four of these additionally had uncontrolled local disease. At a median follow-up time of 28 months (range, 2–68 months), 36 patients (12.2%) have relapsed: 11 of these (30.5%) had local relapse and 25 (69.5%) patients, systemic relapse.

### Toxicity

Overall, treatment was well-tolerated. Toxicity during chemoradiation is shown in Table 4. As expected, the most common toxicities were hematologic and gastrointestinal. There were no episodes of neutropenic sepsis, renal failure, or any other condition directly related with the treatment that required hospitalization.

**Table 4:** Acute toxicity Common Toxicity Criteria National Cancer Institute (CTC NCI) version 2 criteria (294 patients)

Grade	0 N (%)	1 N (%)	2 N (%)	3 N (%)	4 N (%)
Dehydration	283 (96)	4 (1.4)	5 (1.7)	2 (0.8)	0 (0)
Fatigue	150 (52)	70 (24)	74 (25)	0 (0)	0 (0)
Anorexia	263 (90)	26 (9)	5 (1.7)	0 (0)	0 (0)
Diarrhea	191 (65)	36 (12)	61 (21)	6 (2)	0 (0)
Proctitis	246 (84)	41 (14)	4 (1.4)	2 (0.7)	0 (0)
Nausea	257 (87)	120 (41)	132 (45)	5 (1.7)	0 (0)
Vomiting	60 (20)	108 (37)	120 (41)	5 (1.7)	0 (0)
Dysuria	233 (79)	51 (17)	10 (3.4)	0 (0)	0 (0)
Dermatitis	241 (82)	43 (14)	10 (3.4)	0 (0)	0 (0)
Neutropenia	3 (1)	78 (26.5)	124 (42)	89 (30)	0 (0)

### Survival

**Figure 1:**
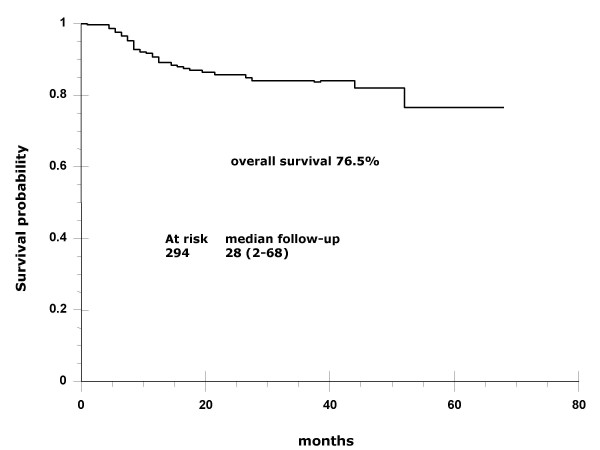
**Overall survival**. Kaplan-Meier curve showing that overall survival at a median 28-month follow-up (range, 2–68 months) was 76.5%.

**Figure 2:**
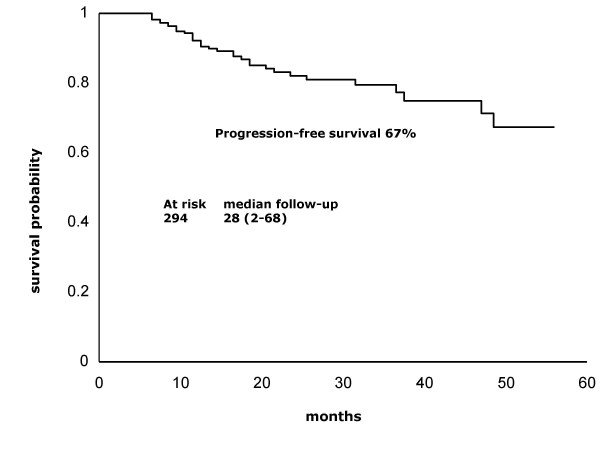
**Progression-free survival**. Kaplan-Meier curve showing that overall survival at a median 28-month follow-up (range, 2–68 months) was 67%.

**Figure 3:**
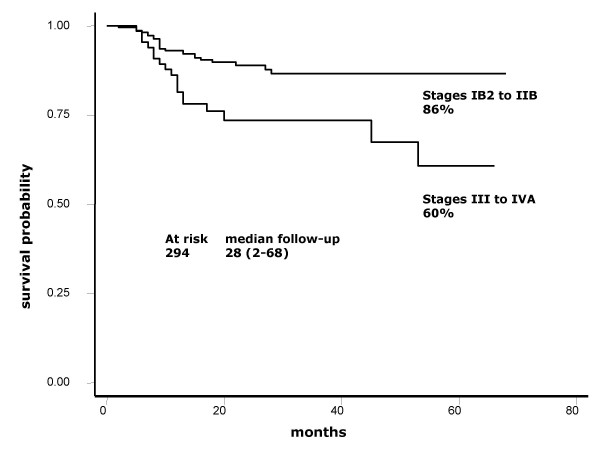
**Overall survival according to FIGO stage**. Kaplan-Meier curve showing overall survival of 86 vs. 60% for earlier stages IB2-IIB against III-IVA.

At a median 28-month follow-up (range, 2–68), overall and progression-free survival are 76.5 and 67%, respectively (Figures 1 and 2). Survival rates according to stages IB2-IIB and IIIB-IVA are 86 vs. 60%, respectively (Figure 3).

## Discussion

Locally advanced cervical cancer remains a significant health problem for many countries in the developing world and for low-income populations of Western countries [2]. Undoubtedly, the greatest efforts should be directed toward improving screening campaigns as the most effective means for reducing cervical cancer mortality; however, while this is being achieved generalized use of the most effective therapies for invasive cancers may contribute to decrease in mortality.

Results of the present report indicate that adding the weekly regimen of cisplatin to standard pelvic radiation in patients outside research settings is achievable, shows no unexpected toxicity, and is effective. At a maximum 68-month follow-up and at a median 28-month follow-up (2–68 months), median survival has not been reached and overall survival is 76.5%. These survival results are very similar to those found in the four randomized studies that formed the foundation for adopting cisplatin-based chemoradiation as the standard of care for locally advanced cervical cancer [3-6]. Table 5 shows 3-year survival as well as patient number and stage distribution in these studies as well as in ours. As can be observed, small differences in the proportion of patients whose disease corresponded to FIGO stages may account for survival variations across the studies. Remarkably, GOG85 and GOG120 studies, which included a higher proportion of FIGO stages IIB and IIIB, had survival percentages within the range of 60–70%, whereas the RTOG9001 study, similar to ours, had more IB and IIB, as reflected in survivals of 75 and 76.5%, respectively. With regard to IB bulky stages, GOG123 reported 83% survival, whereas this was 82% in our IB2 and IIA patients. With respect to toxicity, absence of a uniform classification system for reporting treatment morbidity has resulted in considerable inconsistency in reporting of treatment toxicity and complications of patients with cervical cancer [13]. A recent meta-analysis on toxicity following radiation alone or in combination with chemotherapy for locally advanced cervical cancer confirmed that concurrent chemotherapy increases actoxicity – gastrointestinal and hematologic – as compared with radiation alone [14]. In our study population, grade 4 toxicities were rare (<1%); nonetheless, grade 3 neutropenia was present in nearly one third (30.3%) of patients. This acute toxicity compares similarly with that found in GOG123 and GOG120 studies employing weekly cisplatin; these authors reported grade 3 leukopenia in 18 and 21%, respectively [5,6], whereas our figure regarding neutropenia was 30.3%. Nevertheless, it is noteworthy that no patient had febrile neutropenia or required hospitalization.

**Table 5:** Percentage of 3-year survival in randomized trials in comparison to the present study

Study	*N *patients	IB2%	IIB%	IIIB%	IVA%	OS
GOG123	183	100	0	0	0	83
GOG85	177	0	61	33.9	2.8	67
RTOG9001	195	32	36	27	3	75
GOG120	177/173	0	51.8	42.4	2.6	65
THIS	294	23.2*	53.2	20.4**	0.7	76.5

*23.2% includes IB bulky and IIA; **20.4% includes 2.3% cases of IIIA. GOG85 and GOG120 had highest proportions of IIB and IIIB.

Notwithstanding this, results from other reports on patients treated with cisplatin and radiation in non-research settings are not uniform. A report on acute toxicity from the Addenbrooke Oncological Centre shows that in general, cisplatin-based chemoradiotherapy for carcinoma of the cervix is well-tolerated; of 74 patients, two had grade 4 toxicity (neutropenia and thrombocytopenia, respectively), and another two patients had grade 3 toxicity. Differently from our report, these authors planned only five cycles of cisplatin for the majority of patients, and only five patients with six and seven applications. Regarding treatment compliance, 97.3% of patients completed external radiation in the expected time and 70.2% were administered the planned number of cycles [15]. Different results in terms of compliance and toxicity were reported by Abu-Rustum et al. on 65 women from minorities (African-American, Caucasian, and Hispanic) receiving weekly cisplatin during radiation. Overall, mean whole-treatment total duration was 55 days (median, 51 days), and 19 of 65 (29.2%) patients had incomplete chemotherapy, nine due to hematologic or renal toxicity. Thus, only seven patients (10.8%) received the six cycles of cisplatin; the majority (60%), however, received five applications (16). A third report on this issue found it difficult for patients to comply with cisplatin treatment due to both toxicity and treatment-unrelated causes. In this report, 112 patients with cervical cancer received five planned courses of cisplatin at 40 mg/m^2 ^during external radiation; all but two completed radiotherapy. Nonetheless, 62 patients (55%) did not undergo the planned five cycles of cisplatin due to treatment toxicity (31%) or non-compliance because of delayed administration of the first cycle or omission of a cycle for reasons other than toxicity (21%). The most common side effects resulting in chemotherapy discontinuation included gastrointestinal complications in seven and impaired renal function in five patients [17].

The satisfactory treatment result in terms of effectiveness that we obtained can stem at least partly from the very good therapeutic adherence achieved. Prior to implementation of chemoradiation at our Institution, the rate of patients abandoning treatment was high; it is certainly surprising that despite the fact that combined treatment is more complicated for patients in terms of visits for chemotherapy delivery and for clinical and laboratory examinations, it actually had a positive impact on patient compliance. Prior to chemoradiation, patients were usually seen by the Physician only twice during external radiation and once for brachytherapy programming; in the combined treatment, patients are seen at least seven times by the Medical Oncologist during external radiation. On the other hand, because of cisplatin's well-known nephrotoxicity, we avoid using this drug for patients >70 years of age or for diabetic or hypertensive patients, because these conditions are associated with some degree of sub-clinical renal dysfunction despite having normal serum levels of creatinine and may be associated with other co-morbidities [18-21]. Under these conditions, we routinely employ carboplatin [21] or gemcitabine when creatinine elevation already exists for any reason including obstructive nephropathy [22].

## Conclusion

Our results support use of chemoradiation with six weekly applications of cisplatin at 40 mg/m^2 ^during external radiation for routine management of locally advanced cervical cancer.

## Competing interests

The author(s) declare that they have no competing interests.

## Authors' contributions

LC and MC were in charge of chemotherapeutic management, LR, JH, and AP performed radiation management, JU participated in information compilation and data management, CL-G and DC participated in patient diagnostic evaluation, and AD-G conceived of and wrote the report. All authors participated in the Discussion and critically read the manuscript.

## Pre-publication history

The pre-publication history for this paper can be accessed here:


